# Employees’ support strategies for mental wellbeing during and beyond the COVID-19 pandemic: Recommendations for employers in the UK workforce

**DOI:** 10.1371/journal.pone.0285275

**Published:** 2023-05-05

**Authors:** Kerri Bailey, Johanna Scheutzow, Emily Cooke, Katie Taylor, Francesco Silvestrin, Anna Naumenko, Rebecca Hadley, Adam Huxley, Sonia Ponzo

**Affiliations:** 1 Thrive Therapeutic Software, Warwickshire, England; 2 University of East Anglia, School of Psychology, Norwich, England; 3 University College London, Institute of Epidemiology & Health Care, London, England; 4 University of Hertfordshire, School of Life and Medical Sciences, Hertfordshire, England; 5 University College London, Institute of Health Informatics, London, England; University of the West Indies at Saint Augustine, TRINIDAD AND TOBAGO

## Abstract

Throughout the COVID-19 pandemic, and beyond for many businesses, employees have had to adapt to new ways of working due to disruptions in traditional practices. It is therefore crucial to understand the new challenges that employees are facing when it comes to taking care of their mental wellbeing at work. To that end, we distributed a survey to full-time UK employees (*N* = 451) to explore how supported they felt throughout the pandemic, and to identify whether there are any additional types of support they would like to receive. We also compared employees’ intentions to seek help before versus during the COVID-19 pandemic, and assessed their current attitudes toward mental health. Based on direct employee feedback, our results show remote workers felt more supported throughout the pandemic compared to hybrid workers. We also found that employees who had previously experienced an episode of anxiety or depression were significantly more likely to want extra support at work compared to those who had not. Furthermore, employees were significantly more likely to seek help for their mental health during the pandemic compared to before. Interestingly, the largest increase in intentions to seek help during the pandemic compared to before was with digital health solutions. Finally, we found that the strategies managers have adopted to better support their employees, an employee’s mental health history, and their attitude to mental health all contributed to significantly increasing the likelihood that an employee would disclose a mental health concern to their line manager. We provide recommendations that encourage organisations to make changes to better support their employees, and we highlight the importance of mental health awareness training for both managers and employees. This work is of particular interest to organisations who are looking to tailor their current employee wellbeing offer to a post-pandemic world.

## Introduction

Since the COVID-19 pandemic was declared, many office-working employees were required to adapt to working from home full-time. During 2019, 26.7% of people in UK employment stated they did some work at home [1], which increased to 46.6% in March 2020 [2]. Of these employees, 86% stated they worked from home as a result of the COVID-19 pandemic. This sudden mass shift to remote work represented a global challenge, since many organisations needed time and resources to adjust to new and unknown ways of working. Employees from numerous sectors reported challenges impacting their ability to work from home effectively [3–8], with many reporting struggling with their mental wellbeing during this time [9–14]. Furthermore, front line workers who did not have the option to work remotely, such as those in healthcare settings, faced heightened psychological pressures and worsened mental illness [15]. However, many employers have since adapted to the challenges that were faced at the beginning of the pandemic, and recommendations have been published regarding how to develop effective strategies when working from home both during a time of crisis [16] and beyond [17]. Moreover, further research has provided suggestions for how organisations can support employee wellbeing whilst working from home [18], and those working on the front line [19].

Despite the published recommendations for organisational support, we do not currently know whether employees’ own mental health help-seeking strategies and intentions have changed as a result of the pandemic. Research before the pandemic found general differences in help-seeking intentions among certain populations. For example, adolescents have been found to prefer more informal help from friends and family [20], whereas university students are more likely to seek help online [21]. In terms of employees in the workplace, research has shown that colleagues and managers are more effective in dealing with mental health problems in comparison to family and friends [22, 23]. However, since the pandemic has changed the typical working environment for many employees, help seeking strategies when struggling with mental health may have also changed. During the pandemic, a range of resources were offered to support employees, such as mental wellbeing training programmes and workshops, one-to-one counselling, staff mentoring, financial security programmes, providing a sense of job security, providing individualised support, and electronic communications [16, 24–27]. However, we currently do not know which methods employees prefer to seek help from, and whether their preferences have changed since the pandemic occurred. This is important to understand so organisations can better direct their resources to most effectively support their employees moving forward.

There is also limited evidence surrounding whether employees have actually felt supported by their line manager, and whether they would feel comfortable disclosing a mental health concern to their line manager if they felt their mental health was suffering. The workplace is an effective environment for preventing, detecting, and managing mental health problems [28], and direct support from line managers is known to play a key role in maintaining positive employee wellbeing [29, 30]. It may be the case that employees’ feelings of support differ depending on their working environment, which could influence how comfortable they are discussing their mental health with their line manager. This is important to explore since some organisations will be returning to the office full-time after the pandemic, whereas others do not plan to ever return to a full-time in-office working environment [31]. Therefore, some organisations may need to update and tailor their existing support strategy to the working environment they plan to adopt beyond the pandemic. From an employee perspective, a government survey in 2021 found 85% of UK adults who were working remotely at that time stated they would now prefer a hybrid approach of both home and office work in the future [32]. With these different working environments and changes to working preferences, it is crucial to understand whether each environment allows employees to feel supported and comfortable reaching out to their line manager when needed, and what more could be done to better support employees’ mental health in the respective working environments.

On top of the challenges posed by an ever-changing working environment as a result of the pandemic, it is important to understand what factors may play a role in determining how likely an employee would seek help for mental health in the workplace. Previous research has found, for example, employees’ attitude toward mental health is an impacting factor, whereby negative attitudes (e.g. stigmatising beliefs) are associated with decreases in willingness to seek psychological help [33, 34], and a decreased likelihood to seek counselling is predictive of a negative attitude toward this [35]. If employees who have negative attitudes toward their mental health are less likely to seek help at work, it may be beneficial to prompt employers to allocate mutual aid resources to mental health awareness training (MHAT) programmes or digital health interventions alongside their current provision of offered mental health support. This is of particular importance since research investigating the efficacy of MHAT programmes has typically examined how training a leader can impact employees’ likelihood to use the resources available to them [36–38], as opposed to how training employees could improve their attitudes toward mental health and in turn increase their likelihood to seek help for mental health in the workplace by taking action themselves.

Based on the literature discussed, it is of utmost importance to understand the new challenges employees are facing when it comes to taking care of their mental wellbeing at work. Identifying where changes need to be made will enable us to provide recommendations for employers about how to redirect their resources tailored to a post-pandemic world. With this in mind, the current study answered three main research questions to fill key gaps in existing knowledge.

First, we examined employees’ current intentions to seek help for their mental health via different help-seeking sources, and compared these to their help-seeking intentions before the COVID-19 pandemic. By asking this question, we aimed to assess whether the pandemic has made employees more or less likely to seek help from certain sources. If preferences have changed, then employers may need to update their resources accordingly. Second, we directly asked employees how supported they have felt by their line manager during COVID-19, how comfortable they feel reaching out to their managers for mental health support, and whether there are any ways they believe they could be better supported at work. We asked these questions to identify whether the working environment impacts employees’ feelings of support at work and how comfortable they are with discussing their mental health with line managers. We also aimed to use this knowledge to provide recommendations about what more can be done to provide optimal levels of mental health support in the workplace. Our third objective was to identify any factors that could increase the likelihood of an employee reaching out to their line manager for support if they were struggling with their mental health. Identifying these factors will help to determine where best to allocate resources to encourage employees to actively seek help for their mental health within the workplace.

## Materials and methods

### Participants

Full-time employees working in the United Kingdom, aged between 18–65 years and able to read and understand English were eligible to participate in this study. Participants were recruited using convenience sampling via social media platforms, and via employers by means of the partner organisation (Thrive Therapeutic Software) between the dates of 17^th^ June 2021 - 15^th^ October 2021. Our final sample consisted of 451 participants (291 female, 152 male, 8 prefer not to disclose; note these statistics refer to sex assigned at birth). A sample size calculator confirmed at least 385 participants was sufficient to account for the population size full time UK workers (24.6 million), with 5% margin of error at a 95% confidence level. Participants’ ages ranged the full scale from 18–65 years (*M* = 38, *SD* = 10.13). Table 1 presents the samples’ demographic information. Informed electronic written consent was obtained in accordance with approval from the Ethics Committee of Health, Science, Engineering and Technology at the University of Hertfordshire (Protocol number: LMS/SF/UH/04584). As compensation for their time, participants were given the opportunity to enter themselves into a prize lottery for the chance to win a £100 Amazon Voucher.

**Table 1:** Participant demographics (N = 451).

Variables		Total	
		N	%
Sex assigned at birth	Male	152	33.70
	Female	291	64.52
	Prefer not to say	8	1.77
Gender identity	Male	151	33.48
	Female	292	64.75
	Other	2	0.44
	Prefer not to say	6	1.33
Sexual orientation	Straight / Heterosexual	382	84.70
	Gay or Lesbian	23	5.10
	Bisexual	24	5.32
	Other	3	0.67
	Prefer not to say	19	4.21
Country of residence	United Kingdom	433	96.01
	Other*	18	3.99
Country of origin	United Kingdom	391	86.70
	Other*	60	13.3
Ethnic origin	English, Welsh, Scottish, Irish, British	364	80.71
	Other*	87	19.29
Level of education	CSE or equivalent / GCSE (Grades D—G)	11	2.44
	O-level or equivalent / GCSE (Grades A—C)	68	15.08
	AS/A-level or equivalent	103	22.84
	Degree or equivalent	181	40.13
	Post-graduate degree or equivalent	55	12.20
	Vocational qualifications (e.g. BTEC, NVQ)	30	6.65
	No formal qualifications	3	0.67
Income (before tax)	Less than £20, 000	56	12.42
	£20, 000 to £29, 999	110	24.39
	£30, 000 to £39, 999	104	23.06
	£40, 000 to £49, 999	51	11.31
	£50, 000 to £59, 999	44	9.76
	£60, 000 or more	66	14.63
	Prefer not to say	20	4.43
Residential status	Homeowner	297	65.85
	Private tenant	86	19.07
	Council tenant	16	3.55
	Living with parents	46	10.20
	Other	6	1.33
Marital status	Single	125	27.72
	Married / Civil partnership	187	41.46
	Living with my partner	104	23.06
	Divorced	25	5.54
	Widowed	1	0.22
	Prefer not to say	9	2.00
Number of children	None	260	57.65
	1	73	16.19
	2–4	109	24.17
	More than 4	3	0.67
	Prefer not to say	6	1.33
Exercise per week	Every day	47	10.42
	At least 5 times a week	78	17.29
	At least 3 times a week	150	33.26
	At least once a week	75	16.63
	Occasionally	71	15.74
	Never	30	6.65
Work environment	Remote / Virtual	287	63.64
	Office / Site / In person	97	21.51
	Hybrid (remote and in person)	67	14.86
Size of organisation	0–50	50	11.09
	51–100	20	4.43
	101–500	34	7.54
	501–1000	30	6.65
	1000 +	317	70.29

* note, due to low numbers amongst some of our categories, data were condensed to “other”.

### Materials

A survey was created using SmartSurvey (see S1 File, Preview of survey). The survey was comprised of the demographic information listed above (see Table 1), and further included employment industry, employment position, number of days of absence in the past year due to ill mental health, previous depression or anxiety episodes, and previous access to mental health care services. Lastly, we asked questions relating to three main areas: mental health support from their employer, intentions to seek mental health support, and attitudes toward mental health.

### Section 1

Section one of the survey asked a series of eight questions relating to employees’ thoughts on how supported they have felt by their employer throughout the pandemic in relation to their mental health, and how comfortable they would feel disclosing a mental health concern to their line manager. Information was also collected relating to any additional types of support employees would like to receive, and whether they had previously suffered with a mental health difficulty. Data was collected using Likert scales and yes/no answers. Detailed information about the wording and scoring of these questions can be found in S1 File, Section 1.

### Section 2

Section two of the survey assessed how likely employees would seek help from certain people if they were struggling with their mental health both now, and before the pandemic, using the General Help Seeking Questionnaire [GHSQ; 39, 40]. We included 15 different help-seeking sources which can be seen in Table 2, to be used as a reference when needed. Participants responded on a Likert scale ranging from 1–7 with the options “extremely unlikely”, “unlikely”, “likely” and “extremely likely” placed at points 1, 3, 5, and 7 respectively. The GHSQ has high test-retest reliability (r = .86) and internal consistency (Cronbach’s α = .70). Scoring on the GHSQ is calculated as an individual score from 1–7 for each type of help-seeking intention, whereby a higher score indicates a stronger likelihood to seek help via that source. Participants were also asked whether they had experienced an episode of mental health difficulty over the past 6 months, and before the COVID-19 pandemic occurred using yes/no answers. Further information about this section of the survey can be found in S1 File, Section 2.

**Table 2:** Scores from the GHSQ and IASMHS (N = 451).

GHSQ	During the pandemic	Before the pandemic	Difference
	M *(SD)*	M *(SD)*	M *(SD)*
Q1: Intimate partner (e.g. girlfriend, boyfriend, spouse, de facto)	5.27 *(1*.*84)*	5.00 *(1*.*97)*	.27 *(1*.16)*
Q2: Friend outside of work (not related to you)	4.84 *(1*.*68)*	4.62 *(1*.*79)*	.23 *(1*.16)*
Q3: Friend/colleague at work	3.77 *(1*.*71)*	3.58 *(1*.*77)*	.20 *(1*.25)*
Q4: Parent or relative	3.91 *(2*.*00)*	3.87 *(2*.*05)*	.04 *(*.*97)*
Q5: Line manager/supervisor	3.76 *(1*.*68)*	3.47 *(1*.*75)*	.29 *(1*.24)*
Q6: Mental health professional (e.g. psychologist, counsellor, social worker)	4.63 *(1*.*71)*	4.34 *(1*.*89)*	.29 *(1*.27)*
Q7: Web-Based Counselling	3.70 *(1*.*74)*	3.33 *(1*.*76)*	.37 *(1*.29)*
Q8: Digital self-help solution (e.g. website, app, online peer support community)	4.07 *(1*.*80)*	3.57 *(1*.*86)*	.50 *(1*.41)*
Q9: Mental health app & integrated service (e.g. in-app coaching with mental health professional)	4.08 *(1*.*75)*	3.57 *(1*.*83)*	.50 *(1*.31)*
Q10: Phone helpline (e.g. employee assistance programme, charity)	3.70 *(1*.*81)*	3.35 *(1*.*84)*	.36 *(1*.33)*
Q11: Doctor/GP	4.57 *(1*.*79)*	4.47 *(1*.*92)*	.10 *(1*.*21)*
Q12: Mental health first aider/similar (at work)	3.06 *(1*.*68)*	2.61 *(1*.*62)*	.45 *(1*.12)*
Q13: Minister/religious leader (e.g. Priest, Rabbi, Chaplain)	1.69 *(1*.*28)*	1.63 *(1*.*24)*	.06 *(*.*73)*
Q14: I would not seek help from anyone	2.72 *(1*.*87)*	2.65 *(1*.*93)*	.07 *(1*.*39)*
Q15: I would seek help from another not listed above	2.12 *(1*.*49)*	2.13 *(1*.*52)*	-.02 *(1*.*04)*
IASMHS	M *(SD)*		
Total score	35.08 *(7*.*89)*		
Psychological Openness	12.78 *(3*.*34)*		
Help-Seeking Propensity	11.44 *(3*.*47)*		
Indifference to Stigma	10.86 *(4*.*02)*		

* The difference is significant at p < .001.

### Section 3

Section three of the survey examined participants’ attitude towards mental health using the Inventory of Attitudes towards Seeking Mental Health Services [IASMHS; 41, 42]. The IASMHS assesses the attitudinal factors that influence the seeking of mental health services covering psychological openness, help-seeking propensity, and indifference to stigma (see Hyland et al., 2015 for more information). Participants were asked to indicate the extent to which they agreed or disagreed with each item using a five-point Likert scale ranging from 0 (disagree) to 4 (agree). The IASMHS has high internal consistency (psychological openness Cronbach’s α = .82; help-seeking propensity Cronbach’s α = .76; indifference to stigma Cronbach’s α = .79) and composite reliability (psychological openness ρc = .70; help-seeking propensity ρc = .76; indifference to stigma ρc = .77) for each factor. Scoring on the IASMHS is calculated as a total score whereby a higher score indicates a more positive attitude toward seeking mental health services, and a lower score indicates a more negative attitude. Choosing disagree on the questionnaire items relating to psychological openness and indifference to stigma equated to a positive attitude, as such these questionnaire items were reverse coded [43].

### Procedure

Participants were checked against eligibility criteria and were required to give electronic informed consent before beginning the survey. Any participants who did not meet eligibility criteria were disqualified at this point. Eligible participants proceeded to complete the survey on their electronic device (computer, phone, or tablet) which took no longer than 10 minutes. At the end of the survey, participants were given the opportunity to enter themselves into a prize draw to be compensated for their time, and were given the researchers email address should they have any further questions about the study.

## Results

All data were analysed using the statistical package RStudio 4.2.0.

### Section 1 –Help-seeking intentions

#### Has the pandemic changed how employees would intend to seek help if they were struggling with their mental health?

We examined the likelihood of employees seeking help if they were struggling with their mental health from 15 different sources of help-seeking, and compared their ratings from how they thought they would have sought help before the pandemic compared to during. Given that data was not normally distributed, a non-parametric Friedman’s ANOVA was used to first examine for a main effect of time (median scores from 1–7 for help-seeking intentions before the pandemic vs during). Results found scores for help-seeking intentions during the pandemic (Med = 3.73) were significantly higher than scores for help-seeking intentions before (Med = 3.53): *χ*^*2*^(1) = 53.62, *p* < .001.

A further Friedman’s ANOVA was used to test for a main effect of help-seeking source (median scores from 1–7 for help-seeking intentions for each of our 15 different help-seeking sources; see Table 2), whereby a significant effect was found: *χ*^*2*^ (14) = 1712.7, *p* < .001. The most likely source to seek help will now be listed from most to least likely as follows: Intimate partner (Med = 5.5), Doctor/GP and Friend outside of work (Med’s = 5), Mental health professional (Med = 4.5), Parent or relative, Digital self-help solution, and Mental health app & integrated service (Med’s = 4), Friend/colleague at work, Line manager/supervisor, Web-Based Counselling, and Phone helpline (Med’s = 3.5), Mental health first aider/similar at work (Med = 3), I would not seek help from anyone (Med = 2), and Minister/religious leader and I would seek help from another not listed above (Med = 1).

**Fig 1:**
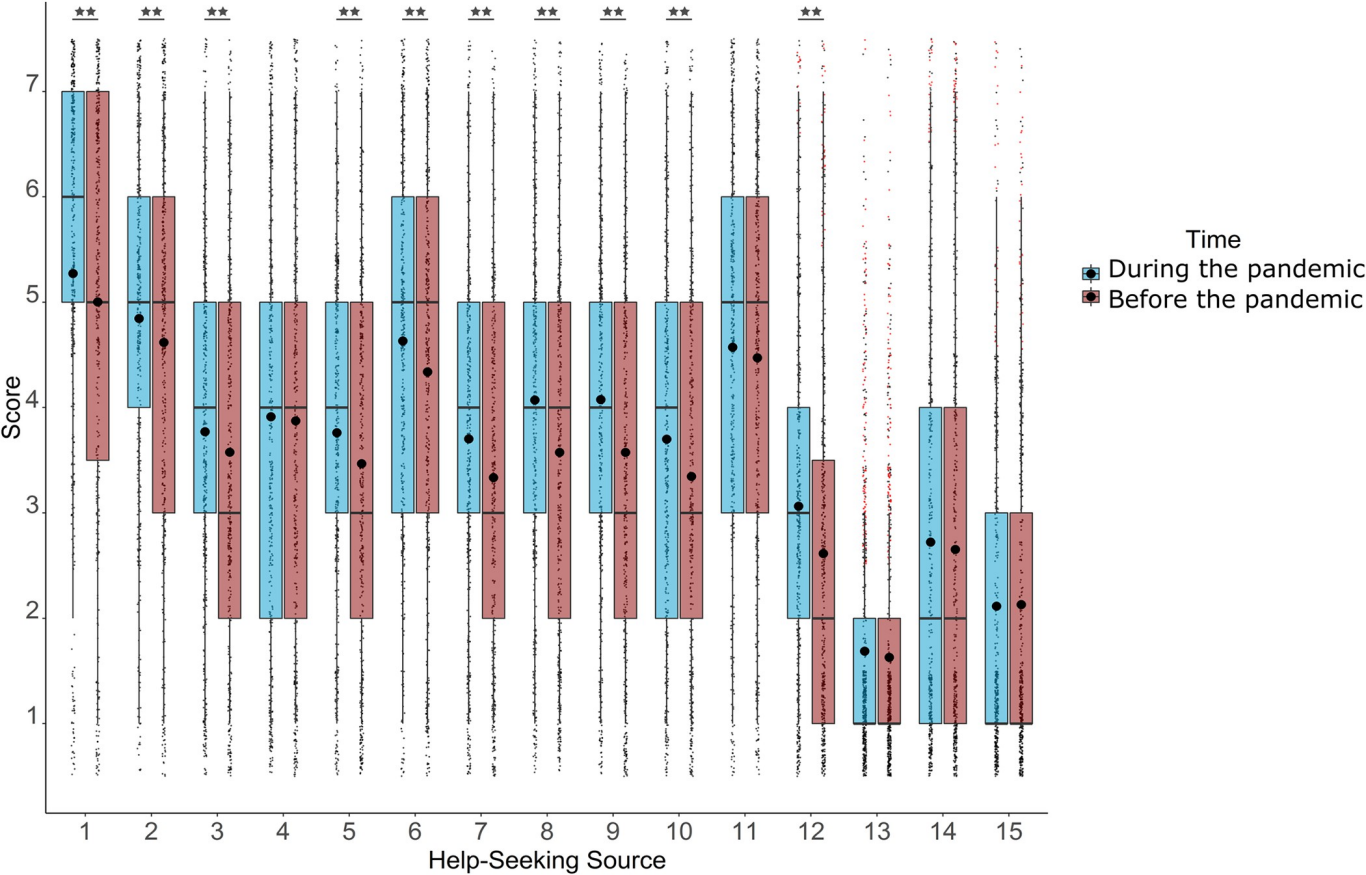
The distribution of scores for participants’ intentions to seek help from each of our 15 different help-seeking sources. Outliers marked as red dots. Help-seeking sources are as follows: 1) Intimate partner, 2) Friend outside of work, 3) Friend/colleague at work, 4) Parent or relative, 5) Line manager/supervisor, 6) Mental health professional, 7) Web-Based Counselling, 8) Digital self-help solution 9) Mental health app & integrated service, 10) Phone helpline, 11) Doctor/GP, 12) Mental health first aider/similar at work, 13) Minister/religious leader, 14) I would not seek help from anyone, 15) I would seek help from another not listed above. □The median. ⚫ The mean. Whiskers: upper whisker = min(max(x), Q_3 + 1.5 x IQR); lower whisker = max(min(x), Q_1–1.5 x IQR). ★★ The difference is significant at Bonferroni corrected ⍺ = .003.

Post-Hoc pairwise Wilcoxon signed rank tests within groups (Bonferroni corrected, *⍺* = .003) revealed 10 help-seeking sources whereby employees were significantly more likely to seek help during when compared to before the pandemic, as can be seen in Fig 1 (see also Table 2).

#### Do employees’ current help-seeking intentions differ depending on the working environment?

We examined the likelihood that employees would currently seek help if they were struggling with their mental health from 15 different sources of help-seeking, and compared their ratings according to work environment (remote, office, hybrid). Checks for normality found the data were not normally distributed, therefore, a robust two-way mixed ANOVA using trimmed means [44] with one within-subjects parameter: help-seeking source (Q1 through to Q15; see Table 2), and one between-subjects parameter: work environment (remote, office, or hybrid) was used to examine whether scores for help-seeking intentions differed between remote, office, or hybrid working employees. Whilst a significant main effect of help-seeking source was found: *F*_14, 310.02_ = 58.212, *p* < .001, we did not find a significant main effect of the work environment (p = .666). However, a significant interaction was found: *F*_28, 320.036_ = 2.112, *p* = .001. Post-Hoc Kruskal-Wallis comparisons examining for differences in work environment for each help-seeking source (Bonferroni corrected, *⍺* = .003) revealed one significant effect (minister or religious leader; *H*_*2*_ = 11.591, *p* = .003), whereby a follow up Wilcoxon Rank-Sum Test revealed remote workers were significantly less likely to seek help from a minister or religious leader compared to office workers (*p* = .003; Bonferroni corrected *⍺* = .017).

### Section 2 –Employee support during and beyond the pandemic

#### Does the amount of support employees feel they have received differ depending on the working environment?

The data for this analysis were not normally distributed, therefore two Kruskall Wallis tests were performed. Results revealed no significant difference in scores for how comfortable employees felt disclosing a mental health concern to their line manager depending on whether they were remote, office, or hybrid working (*H*_*2*_, = 1.517, *p* = .468). However, a significant difference in scores was found for the amount of support employees felt they had received from their line manager during the pandemic, according to the working environment (*H*_*2*_ = 6.89, *p* = .032). A follow up Wilcoxon Rank-Sum Test revealed remote working employees felt significantly more supported by their line manager during the COVID-19 pandemic compared to hybrid working employees (*p* = .010; Bonferroni corrected *⍺* = .017).

#### Does the frequency of conversations about mental health relate to employees feeling better supported and more comfortable reaching out to their line manager?

**Fig 2:**
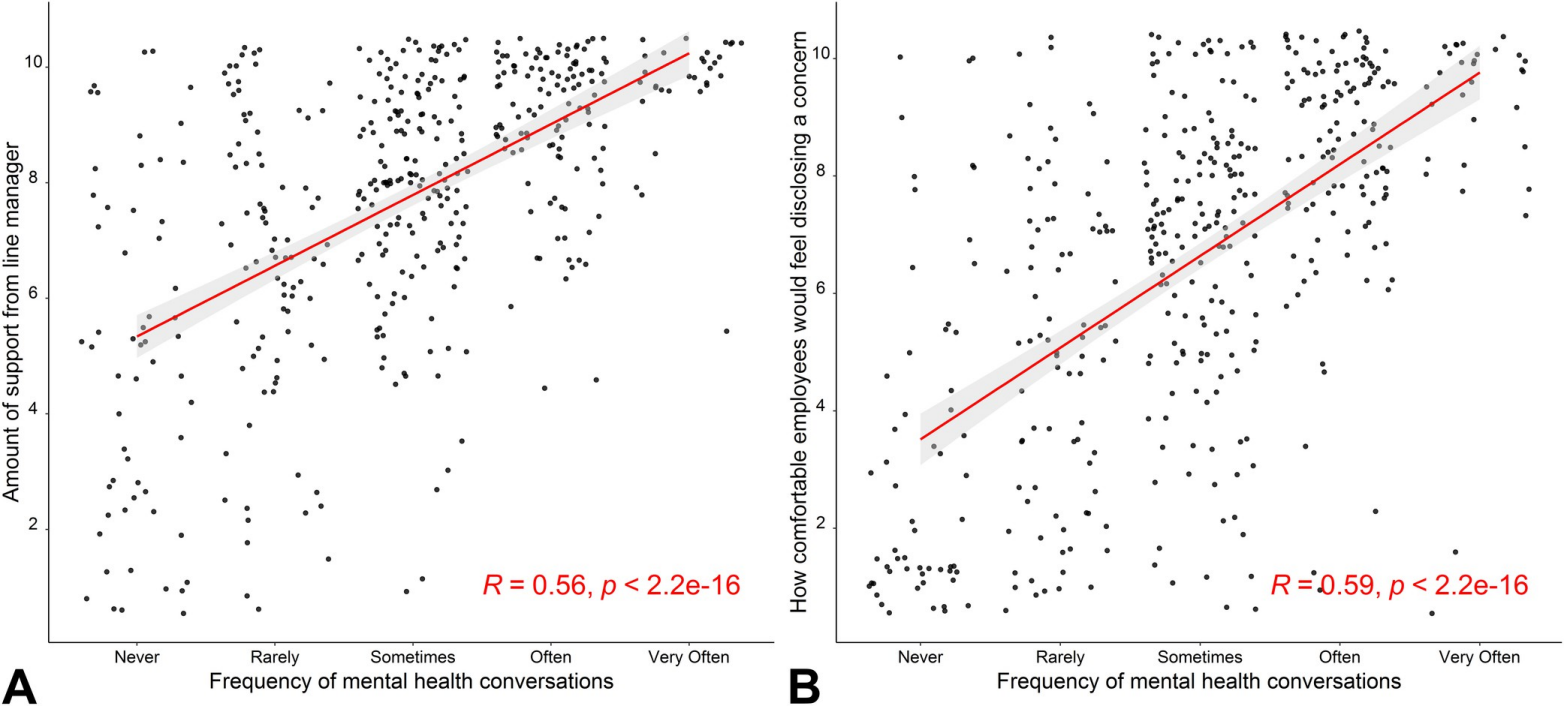
The relationship between the frequency of conversations about mental health encouraged by the line manager with: (A) The amount of support employees feel they had from their line manager, and (B) How comfortable employees would feel disclosing a mental health concern to their line manager. Both scatter plots display a positive correlation, meaning the more mental health conversations that are encouraged by the line manager, the more supported employees feel in the workplace, and the more comfortable they would feel disclosing a mental health concern to their line manager.

A Spearman’s correlation, run to assess the relationship between the amount of support employees felt they had received during the COVID-19 pandemic and the frequency of conversations about mental health and wellbeing encouraged by the line manager, revealed a statistically significant positive correlation between the two variables (*r*_*s*_ = .56, *p* < .001; Fig 2A). A second Spearman’s correlation, conducted to assess the relationship between how comfortable employees would feel disclosing a mental health concern to their line manager and the frequency of conversations about mental health encouraged by the line manager, revealed a significant positive correlation (*r*_*s*_ = .59, *p* < .001; Fig 2B).

#### What factors are associated with employees wanting to receive different types of extra support?

Multiple chi-square analyses were conducted to determine whether the type of extra support employees would like their line manager / immediate supervisor at work to provide (e.g. offer more flexible working, make time to discuss concerns or issues) was associated with any demographic, organisation, or mental health related factors. Due to low sample sizes in some of our response options, data binning was used to create fewer categories for these analyses (see S1 Table for further details). Differences in sample sizes are due to filtering out options such as “prefer not to say” in some of our analyses. Only analyses that survived Bonferroni corrections for multiple comparisons are reported.

*Extra support option 1*: *Encourage open conversations about wellbeing and mental health at work*

In terms of employees who stated they would like their line manager to encourage more open conversations about wellbeing and mental health at work, chi square analysis revealed a significant association whereby 55.24% of employees who had previously experienced depression or anxiety episodes stated they would like more conversations, compared to 37.36% of employees who stated they would like more conversations but had not experienced a previous episode of depression or anxiety: *χ*^*2*^ (1, N = 451) = 13.68, *p* < .001.

*Extra support option 2*: *Offer flexible working*

No factors were found to be significantly associated with employees who stated they would like their organization to offer more flexible working.

*Extra support option 3*: *Make time for me to discuss concerns or issues*

Chi square analyses revealed a significant association for previous depression or anxiety episodes: *χ*^*2*^ (1, N = 451) = 10.15, *p* = .001, whereby 35.38% of employees who had experienced previous anxiety or depression episodes stated they would like their organization to make more time to discuss concerns or issues compared to 21.26% of employees who would like their organization to make more time to discuss concerns or issues but had not previously experienced an episode of anxiety or depression.

*Extra support option 4*: *Make adjustments to help me manage demands of my work*

No factors were found to be significantly associated with employees who would like their organization to make adjustments to help them manage the demands of their work.

#### Section 3 –Which factors predict the likelihood that an employee will disclose a mental health concern to their line manager?

To assess which factors contribute to the likelihood that an employee would seek help from their line manager if they were struggling with their mental health, we ran a hierarchical linear multiple regression with the criterion variable being the likelihood that an employee would seek help from their line manager/supervisor if they were currently struggling with their mental health (Q5 in the help-seeking intentions questionnaire with response scores from 1–7; see Table 2), and two hierarchical steps for the predictor variables. Step 1 included 9 predictors, which related to the individual’s attitude to mental health, an employee’s mental health history, and anything the organisation and/or line manager has done to help employees feel supported (see S2 Table). Note the mean score for the IASMHS was 35.08 (*SD* = 7.89, range = 11–48 from a total possible range of 0–48; see Table 2 for further details) for our participants. Step 2 added in demographic and work related factors, resulting in an additional 21 predictors, to determine whether any of these factors also contributed to predicting how likely an employee would seek help from their line manager if they were struggling with their mental health (see S2 Table). This resulted in 30 predictors total across our two hierarchical steps.

Results showed a significant model at step 1, which explained 43.93% of the variance (*F*_9, 406_ = 37.12, *p* < .001), in which five of our nine variables were significant predictors as follows: How supported employees felt by their line manager during the COVID-19 pandemic (*β* = .19, *t* = 5.63, *p* < .001), how often their line manager encourages conversations about an employee’s mental health and wellbeing (*β* = .40, *t* = 5.56, *p* < .001), how much an employee feels their organisation has provided sufficient support for their mental health during the COVID-19 pandemic (*β* = .38, *t* = 5.26, *p* < .001), how many days absence an employee has had due to mental health (*β* = .37, *t* = 2.69, *p* = .007), and an employee’s attitude to mental health (*β* = .02, *t* = 2.48, *p* = .014). All five of these variables were positively related to an employee’s likelihood to seek help from their line manager if they were struggling with their mental health.

Then, we added a second step to the regression whereby we included a further 21 predictor variables in addition to the previous 9 predictor variables, to examine whether any demographic or work-related factors were also significant predictors of an employee’s likelihood to seek help from their line manager if they were struggling with their mental health. The regression remained significant, explaining 45.20% of the variance (an additional 1.27%; *F*_27, 388_ = 13.68, *p* < .001). However, when running an ANOVA to assess whether the change in R2 was significantly improved at step 2, the additional demographic and work-related factors did not significantly improve the fit of the model to the data (*F*_18, 388_ = 1.52, *p* = .078).

Finally, since the data were not normally distributed and transformations did not improve the shape of the distribution, we ran an ordinal logistic multiple regression on the data (untransformed). The reason for this analysis was to examine whether we could corroborate the findings from the hierarchical linear multiple regression at step 1 above whilst assuming no linearity by changing the criterion variable to ordinal data (the likelihood that an employee would seek help from their line manager/supervisor if they were currently struggling with their mental health from 1–7). The model included our 9 predictors from step 1 relating to the individual’s mental health, and anything the organisation and/or line manager has done to help employees feel supported. Results corroborated the hierarchical linear multiple regression above, with the same five predictors being significant at *p* ≤ .008. Calculated odds ratios for an example employee who scores the highest on all our predictors (how supported employees felt by their line manager during the COVID-19 pandemic = 10, how often their line manager encourages conversations about an employees mental health and wellbeing = 5, how much an employee feels their organisation has provided sufficient support for their mental health during the COVID-19 pandemic = 5, how many days absence an employee has had due to mental health = 1+, and an employee’s attitude to mental health = 48) showed that there would be a 94.2% chance of scoring 5 or more on the likelihood that they would seek help from their line manager if they were struggling with their mental health.

## Discussion

This study found full-time UK employees have significantly higher intentions to seek help if struggling with their mental health now (during the COVID-19 pandemic), compared to before the pandemic occurred. Whilst we did not find significant differences in employees’ help-seeking intentions between different working environments, we did identify where employers need to focus their resources in order to better support their employees at work. Specifically, we found that employees who had previously experienced an episode of anxiety or depression were significantly more likely to want extra support at work compared to those who had not. Finally, we found five factors that significantly increased the likelihood that an employee would reach out to their line manager for support if they were struggling with their mental health; employee’s attitude towards mental health, an employee’s mental health history, and strategies that managers have adopted to better support their employees. Thus we provide recommendations to the UK workforce about where resources could be best allocated to encourage employees to seek help for their mental health within the workplace. For example, MHAT programmes for both managers and employees should, based on our data, increase the likelihood that an employee will disclose a mental health concern at work.

*How the pandemic has changed employees’ intentions to seek help if they were struggling with their mental health*

We found that employees had significantly higher intentions to seek help if they were struggling with their mental health during the COVID-19 pandemic, when compared to how they felt they would have sought help before. Interestingly, previous research during the early stages of the pandemic found people were reluctant to seek help for their mental health since they did not believe it was a priority during that time [45–47]. This is in contrast with our findings; one possibility is that people may be more likely to seek help for a mental health problem since moving past the initial crisis stage of the pandemic. Furthermore, over the past year recommendations about how to develop effective support strategies during a time of crisis [16] and beyond [17] have been published. As such, organisations and managers may have only more recently made appropriate changes to better support their employees’ mental wellbeing. We know from our current research that this is an influencing factor, since we found a significantly positive relationship between the frequency of conversations about mental health encouraged by the line manager and how comfortable employees felt reaching out to their line manager to discuss a mental health concern.

When exploring differences in specific help-seeking sources, digital solutions (e.g. web-based counselling, phone helplines, and mental health apps with integrated services) revealed the biggest difference scores in help-seeking intentions from before the pandemic to during, with employees being more likely to utilise this type of support now. The reason for this large increase could be due to the fact that digital solutions were largely the only accessible option for many people at that time. On the other hand, it may be the case that the pandemic enabled people to become aware of options they did not previously know were available to them, which they now intend to use. Nevertheless, this information is valuable to the UK workforce moving forwards, since research has argued that digital solutions should be scaled up and used more effectively to decrease the mental health burden that the COVID-19 pandemic caused [48]. We therefore recommend that organisations provide their employees with access to digital self-help solutions in the workplace, such as mental health apps [49–51]. It is crucial that these apps hold a strong evidence base with proven efficacy, so as to ensure the workplace is promoting effective treatment for their employees who are struggling with their mental health [52, 53].

Interestingly, we found no significant change in help-seeking intentions through a doctor or GP from before the pandemic to now. Since help through a GP is a very common initial point of contact even before the pandemic [54], we suggest employees aim to continue seeking help from sources they had prior knowledge about before the pandemic occurred, in addition to new sources (e.g. digital mental health solutions). It is important to note a limitation of this study is that we only retrospectively asked our participants about their help-seeking intentions before the COVID-19 pandemic occurred, which could present some bias in participant answers and hence should be interpreted with this in mind. Furthermore, it is worth noting that help-seeking intentions may not represent actual help-seeking behaviour. Future research could explore whether an employee’s current help-seeking intentions matches up to their future behaviour in a longitudinal study.

*Recommendations for how organisations can redirect their resources to better support their employees beyond the pandemic*

We found that employees working remotely felt significantly more supported by their line manager compared to employees who were hybrid working. The reason for this could be that managers of employees who were forced into a fully remote working environment may have made more effort to ensure their employees were supported during that time, given the drastic change to their working environment and lack of face to face contact with their colleagues. Future research should compare remote workers feelings of support from their line manager both pre- and post- pandemic to examine whether this is true. Interestingly, recent research found working exclusively remotely had a negative effect on employee wellbeing in terms of workplace relationships [55], which suggests whilst employees working remotely felt more supported by their immediate line manager or supervisor (perhaps due to regular scheduled online meetings), they felt disconnected from the rest of the workplace due to the remote environment. We therefore recommend that remote working organisations should encourage more integration amongst colleagues to enhance employee wellbeing beyond the direct support they receive from their line manager, whilst ensuring regular meetings with their manager still occur. For example, introducing wellbeing champions, who are employees of the company dedicated to supporting the wellbeing of staff, is an excellent way to help employees feel more connected when working remotely [56].

We found those who were hybrid working felt significantly less supported by their line manager compared to remote workers. Since our data revealed a significant positive relationship between the frequency of conversations about mental health encouraged by the line manager and how supported employees felt during the pandemic, we recommend hybrid working organisations should prioritise having regular conversations about mental health with their workforce to help them feel better supported at work. Hybrid organisations should also support flexible working arrangements by giving the employee the option to choose their preferred format of the meeting (online or face to face). Furthermore, managers should hold an understanding that specific days working at home or in the office are not fixed and could vary, and therefore be as accommodating as possible for this. This is particularly important to the many organisations who have recently adopted a permanent hybrid workforce [31], since their existing wellbeing strategies are likely tailored to the wrong working environment.

We also asked employees whether there were any particular types of extra support they would like to receive in the workplace, and with this data we examined whether any specific demographic, work, or mental health related factors were associated with certain types of extra support needed. This allowed us to identify where organisations may need to make changes to better support their employees. We found that employees who had experienced a previous depression or anxiety episode would like their manager to encourage more conversations about mental health and wellbeing at work, and make more adjustments to help them manage demands with their work. These findings may be due to a lack of understanding of mental health conditions from the manager. Previous research found that training managers in workplace mental health improves their knowledge and attitude to addressing any mental health concerns, and self-reported behaviour in supporting their employees who experience mental health problems [38]. As such we recommend all managers receive evidence-based mental health training in order to effectively support their employees. This should also help to foster a positive atmosphere which could encourage employees to share their mental health history or current issues experienced, allowing managers to make reasonable adjustments.

It is important to highlight the limitation that our survey questions were closed, and future research should consider taking a qualitative approach to better understand the extra support employees would like to receive. For example, the specific needs for flexible working or reasonable adjustments would be very different for an employee working in computer and technology compared to an employee working in health care. Therefore, obtaining an in-depth understanding about what their specific needs are when they state the type of extra support they would like to receive is a vital area of research that needs to be explored. Furthermore, we addressed the limitation of small sample sizes in some of our demographic categories by using data binning in any analyses that used this information. However, it is important to note a limitation of our study is that our data may lack generalisability due to small samples in some categories. For example, our sample consisted of more people who had no children, compared to those who did. Research has shown that people with children experienced higher mental health distress as a result of the pandemic in comparison to people who do not have children [57]. This increase in mental health distress could have further increased their likelihood to seek help if they were struggling with their mental health during the pandemic when compared to before. It would be an interesting avenue for future research to investigate differences in increased help-seeking intentions amongst different demographic populations such as this.

*What managers can do to increase the likelihood that an employee will seek support from them when struggling with their mental health*

Our study found factors related to an employee’s mental health history, an employee’s attitude to mental health, and strategies that managers have adopted to better support their employees, significantly contributed to predicting the likelihood that an employee would seek help via their line manager if they were struggling with their mental health. These factors will act as key recommendations for the UK workforce since we already know from previous research that the workplace is an effective context for preventing, detecting, and managing mental health problems [28], with evidence-based training and direct support from line managers playing a key role in maintaining positive employee wellbeing [29, 30, 58]. We found an employee’s intentions to reach out for help via their line manager was significantly increased if they felt their line manager had provided sufficient support for them throughout the COVID-19 pandemic, and if their line manager encouraged regular conversations about their mental health and wellbeing. Therefore, we encourage managers to promote more conversations about mental health in the workplace to enhance the likelihood that employees will reach out to them. This could be achieved by scheduling regular meetings to specifically discuss mental health. Managers could also be provided with the mental health training they may need, to increase their mental health knowledge, enhance their attitudes to mental health, and improve their behaviour [38]. We also found employee’s own attitudes to mental health were a significant predictor of their likelihood to disclose a mental health concern, which is in line with previous research observing negative attitudes about mental illness are associated with decreases in willingness to seek psychological help [33, 34]. For this reason, organisations should offer mental health training courses for all employees to encourage positive attitudes to mental health, which should in turn increase the likelihood that they will reach out to their line manager if they need to. The final significant predictor of the likelihood to disclose a mental health concern was that of employees who had previously experienced one or more days absence due to mental health, which suggests that employees who have previously disclosed a mental health concern to their line manager intend to do so again if they were to experience another mental health concern in the future.

## Conclusion

In summary, this study found that employees have significantly higher intentions to seek help when struggling with their mental health during, compared to before, the COVID-19 pandemic. Specifically, employees reported significantly higher intentions to seek help from digital health solutions, whereas they are no more likely to seek help from a doctor or GP. Furthermore, we found differences in the types of extra support employees would like to receive, whereby employees who had previously experienced an episode of anxiety or depression were significantly more likely to want extra support at work compared to those who had not. Thus we provide key recommendations for the UK workforce to adopt whilst taking an employee’s mental health history into account. Finally, we found an employee’s mental health history, their attitude to mental health, and support strategies that managers have adopted for their employees all significantly increased the likelihood that an employee would disclose a mental health concern to their line manager. We advise managers and supervisors of employees in the UK workforce to reflect on our recommendations and make reasonable adjustments to their current employee wellbeing offer in order to better support their employees’ mental health and wellbeing.

## Supporting information

S1 Table Condensed data for the chi square analysis. (DOCX)

S2 Table Predictors inputted into the regression analysis. (DOCX)

S1 File Supplementary information. (DOCX)
