# Supplementary material for: Employees’ support strategies for mental wellbeing during and beyond the COVID-19 pandemic: Recommendations for employers in the UK workforce
Source: PLoS One. 2023 May 5;18(5):e0285275. doi: 10.1371/journal.pone.0285275 (PMC10162522; doi:10.1371/journal.pone.0285275)
Supplement: S1 Table — (DOCX) [file pone.0285275.s001.docx]

**S1 Table. Condensed data for the chi square analysis.**

| **Variables** |  | **Total** | |
| --- | --- | --- | --- |
|  |  | **N** | **%** |
| Age | 18-35 | 212 | 47.01 |
|  | 36-50 | 174 | 38.58 |
|  | 51-65 | 65 | 14.41 |
| Sex at birth | Male | 152 | 34.31 |
|  | Female | 291 | 65.69 |
| Gender identity | Male | 151 | 34.09 |
|  | Female | 292 | 65.91 |
| Sexual orientation | Straight / Heterosexual | 382 | 84.70 |
|  | Gay / Lesbian/ Other | 69 | 15.30 |
| Country of origin | United Kingdom | 391 | 86.70 |
|  | Other | 60 | 13.30 |
| Ethnic origin | English, Welsh, Scottish, Irish, British | 364 | 80.71 |
|  | Other | 87 | 19.29 |
| Education | University degree | 236 | 52.33 |
|  | No university degree | 215 | 47.67 |
| Income (before tax) | Less than £30,000 per year | 166 | 38.52 |
|  | £30,000 to less than £50,000 per year | 155 | 35.96 |
|  | £50,000 + per year | 110 | 25.52 |
| Residential status | Home owner | 297 | 68.85 |
|  | Rent, living with parents, other | 154 | 34.15 |
| Marital status | Married / civil partnership / living with partner | 291 | 64.52 |
|  | Single / divorced / widowed | 151 | 33.48 |
| Number of children | None | 260 | 58.43 |
|  | 1 + | 185 | 41.57 |
| Exercise per week | 3 times or more per week | 275 | 60.98 |
|  | Less than 3 times per week | 176 | 39.02 |
| Work environment | Remote/ virtual | 287 | 63.64 |
|  | Office / on site / in person | 97 | 21.51 |
|  | Hybrid (remote and in person) | 67 | 14.86 |
| Size of organisation | 500 or less | 106 | 23.06 |
|  | More than 500 | 347 | 76.94 |
| Industry role | Management role | 204 | 45.23 |
|  | Non-management role | 247 | 54.77 |
| Days of absence due to mental health | None | 306 | 67.85 |
|  | 1 or more | 145 | 32.15 |
| Previous depression or anxiety episodes | Yes | 277 | 61.42 |
|  | No | 174 | 38.58 |
| Previous access to mental healthcare services | Yes | 206 | 45.68 |
|  | No | 245 | 54.32 |

* note, due to low numbers amongst some of our categories, data were condensed to “other”.
