# Supplementary material for: Employees’ support strategies for mental wellbeing during and beyond the COVID-19 pandemic: Recommendations for employers in the UK workforce
Source: PLoS One. 2023 May 5;18(5):e0285275. doi: 10.1371/journal.pone.0285275 (PMC10162522; doi:10.1371/journal.pone.0285275)
Supplement: S2 Table — (DOCX) [file pone.0285275.s002.docx]

**S2 Table. Predictors inputted into the regression analysis.**

| **Predictors** |  |
| --- | --- |
| **Step 1** |  |
| Factors related to an employees’ attitude to mental health, and an employees’ mental health history | Previous depression or anxiety episodes |
|  | Mental health suffered as a result of the pandemic |
|  | Mental health difficulty in the past 6 months |
|  | Mental health difficulty before the COVID-19 pandemic occurred |
|  | Number of days absence due to mental health |
|  | Attitude to mental health |
| Factors related to things the organisation and/or line manager has done to help employees feel supported | How often line manager encourages conversations about mental health |
|  | How supported employees have felt by their line manager during the COVID-19 pandemic |
|  | Whether the organisation has provided sufficient support for their mental health during the COVID-19 pandemic |
| **Step 2** |  |
| Employee demographic factors | Age 18 - 35 |
|  | Age 36 - 50 |
|  | Age 51 - 65 |
|  | Sex at birth |
|  | Gender identity |
|  | Sexual orientation |
|  | Country of origin |
|  | Level of education |
|  | Income less than £30,000 per year |
|  | Income £30,000 to less than £50,000 per year |
|  | Income £50,000 + per year |
|  | Residential status |
|  | Marital status |
|  | Number of children |
|  | Amount of exercise per week |
| Work related factors | Remote working |
|  | Office working |
|  | Hybrid working |
|  | Size of organisation |
|  | Industry role |
