# Supplementary material for: Employees’ support strategies for mental wellbeing during and beyond the COVID-19 pandemic: Recommendations for employers in the UK workforce
Source: PLoS One. 2023 May 5;18(5):e0285275. doi: 10.1371/journal.pone.0285275 (PMC10162522; doi:10.1371/journal.pone.0285275)
Supplement: S1 File — (DOCX) [file pone.0285275.s003.docx]

**Supplementary information**

**Preview of survey:** <https://www.smartsurvey.co.uk/s/preview/employeewellbeingsurvey/2DED72D02F73AC2E8B736228FF39B5>

**Questions asked:**

**Demographic**

Age, sex assigned at birth, gender identification, sexual orientation, country of residency, country of origin, ethnicity, level of education, gross yearly income before tax, residential status, marital status, number of children, and sedentary/active lifestyle. Furthermore, information regarding each employee's working environment was collected, in addition to the size of organisation, employment industry, employment position, number of days of absence in the past year due to mental health, previous depression or anxiety episodes, and previous access to mental health care services.

**Section 1**

Section one of the survey asked a series of eight questions relating to employees' thoughts on how supported they have felt by their employer throughout the pandemic in relation to their mental health. These eight questions are detailed as follows: 1) “How often does your line manager encourage conversations about your mental health and wellbeing?” with the options “never”, “rarely”, “sometimes”, “often” and “very often”. 2) “On a scale of 1 - 10, how comfortable would you feel disclosing mental health concerns with your line manager?” where 1 = “not comfortable”, and 10 = “comfortable”. 3) “On a scale of 1 - 10, how supported have you felt by your line manager during COVID-19?” where 1 = “not supported”, 10 = “supported”. 4) “Thinking about how my manager/immediate supervisor supports me at work, I want them to do more of the following:”. Participant were given multiple choice options with the following answers: “Encourage open conversations about wellbeing and mental health at work”, “Offer flexible working”, “Make time for me to discuss concerns or issues”, “Make adjustments to help me manage demands of my work”, “None of the above”. 5) “In the last 12 months, have you accessed any support for your mental health?” With the options “yes” and “no”. 6) “If yes, how did you access support?” with the options “Through my employer (e.g. EAP, digital counselling)”, “Privately (e.g. personal private medical insurance, private counselling)”. “Via the National Health Service or local equivalent (e.g. GP, community mental health services)”, “N/A”, and “Other (please specify)”. 7 and 8) “My mental health has suffered as a result of the pandemic” and “My organisation has provided sufficient support for my mental health during the pandemic”, both with the options “strongly disagree”, “disagree”, “neither agree not disagree”, “agree” and “strongly agree”.

**Section 2**

In accordance with recommendations by Ajzen [40], we used the standard wording in which the problem-probe “mental health” was used, meaning the wording of our questionnaire was as follows: “If you were struggling with your mental health, how likely is it that you would seek help from the following people?”. When asking the same question in relation to before the pandemic, participants were asked whether they had experienced an episode of mental health difficulty BEFORE the COVID-19 pandemic occurred. Then, the wording for the GHSQ was adapted to: “BEFORE the COVID-19 pandemic occurred, if you were struggling with your mental health, how likely is it that you would seek help from the following people?”. Help sources were determined based upon the existing questionnaire structure and consultation with both the Clinical Research team and the Business Psychology team at Thrive Therapeutic Software. This resulted in 15 help-seeking intentions which can be seen in Table 1 of the main text, to be used as a reference when needed. Participants responded on a Likert scale ranging from 1 - 7 with the options “extremely unlikely”, “unlikely”, “likely” and “extremely likely” placed at points 1, 3, 5, and 7 respectively.

**References**

40. Ajzen I. Constructing a TPB questionnaire: Conceptual and methodological considerations. accessed 28/12/2007. 2002.
